# Complex hybrid management of severe aortic stenosis and aortic arch disease in a nonagenarian: a case report

**DOI:** 10.1093/ehjcr/ytag161

**Published:** 2026-03-12

**Authors:** Mirko Muretti, Maria Antonella Ruffino, Giovanni Pedrazzini, Stefanos Demertzis, Enrico Ferrari

**Affiliations:** Cardiac Surgery Unit, Cardiocentro Ticino Institute, EOC, Via Tesserete 48, 6900 Lugano, Switzerland; Radiology Department, Canton of Ticino, Cantonal Hospital Group, Via Tesserete 46, 6900 Lugano, Switzerland; Cardiology Unit, Cardiocentro Ticino Institute, EOC, Via Tesserete 48, 6900 Lugano, Switzerland; Faculty of Biomedical Sciences, Università della Svizzera Italiana (USI), Campus est, Via la Santa 1, 6900 Lugano, Switzerland; Cardiac Surgery Unit, Cardiocentro Ticino Institute, EOC, Via Tesserete 48, 6900 Lugano, Switzerland; Faculty of Biomedical Sciences, Università della Svizzera Italiana (USI), Campus est, Via la Santa 1, 6900 Lugano, Switzerland; Faculty of Medicine, University of Bern, Murtenstrasse 11, 3008 Bern, Switzerland; Cardiac Surgery Unit, Cardiocentro Ticino Institute, EOC, Via Tesserete 48, 6900 Lugano, Switzerland; Faculty of Biomedical Sciences, Università della Svizzera Italiana (USI), Campus est, Via la Santa 1, 6900 Lugano, Switzerland; Faculty of Medicine, University of Zurich (UHZ), Künstlergasse 15, 8001 Zurich, Switzerland

**Keywords:** Case report, Thoracic aortic aneurysm, Aortic valve stenosis, Combined procedure, Thoracic endovascular aortic repair, TEVAR, Transcatheter aortic valve replacement, TAVR, Aortic arch debranching

## Abstract

**Background:**

Nonagenarians represent a cohort of patients at high or prohibitive risk in case of complex aortic procedures. A hybrid approach could reduce the surgical risk compared with a conventional surgery when performed at the age of 90.

**Case summary:**

We reported the case of a 90-year-old man who presented with a progressive aortic arch dilatation due to a persistent type-1A endoleak following previous thoracic endovascular aortic repair due to type-B aortic dissection. The aortic computed tomography scan confirmed a 65 × 67 mm aortic arch dilatation due to a type-1A endoleak and a transthoracic echocardiogram showed a severe aortic valve stenosis. On full sternotomy and partial cardiopulmonary bypass assistance, the patient underwent transcatheter aortic valve replacement combined with surgical brachiocephalic trunk and left common carotid artery debranching and thoracic endovascular aortic repair of the aortic arch. The postoperative course was uneventful and the patient was discharged home at postoperative day 15.

**Discussion:**

The described hybrid procedure lessened the prohibitive risks that could have been related to a conventional open aortic arch surgery in nonagenarians.

Learning points Concomitant TAVR, brachiocephalic trunk and left common carotid artery debranching plus TEVAR in zone 0 is a hybrid procedure able to reduce the otherwise prohibitive risk of a complex operation in nonagenarians

## Introduction

The number of nonagenarians is constantly growing and they represent a cohort of patients at high or prohibitive risk in case of complex aortic procedures. The use of combined transcatheter and surgical techniques can help reducing the surgical risk and postoperative complications.

## Summary figure

**Figure:**
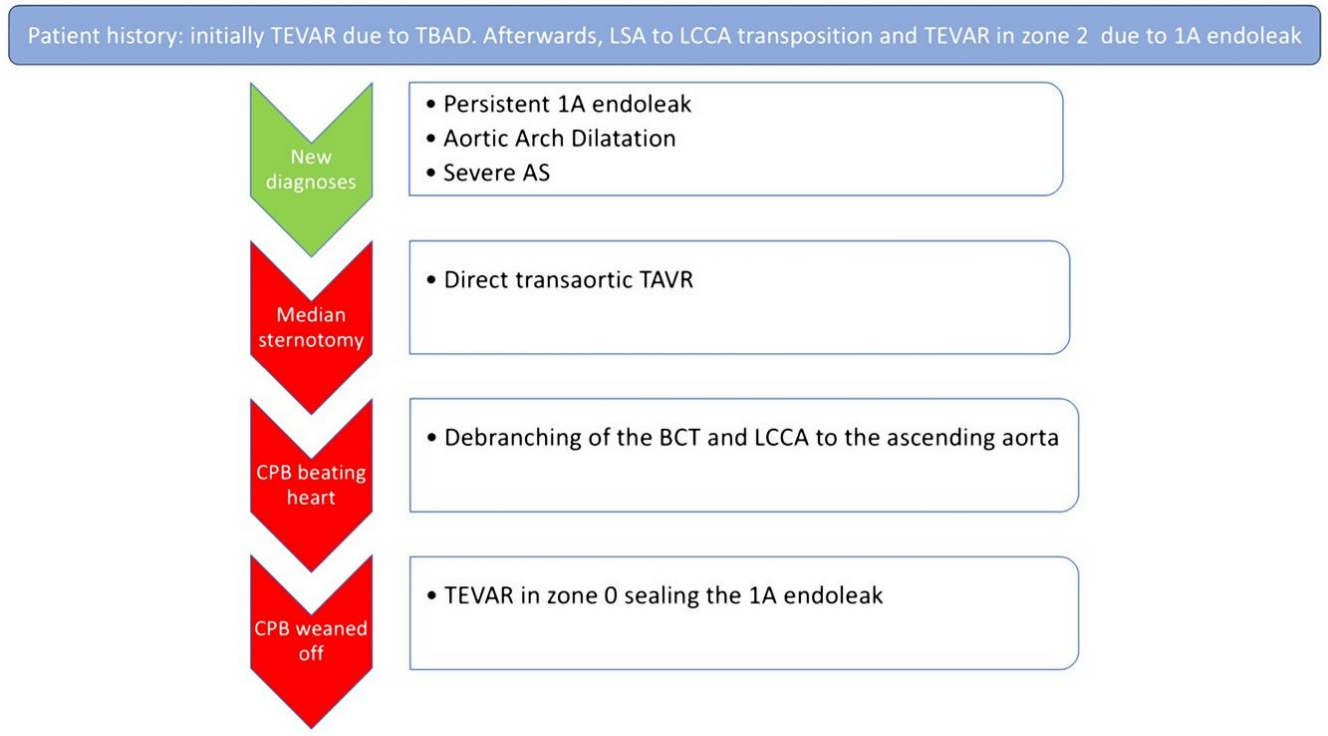


## Case presentation

**Figure 1:**
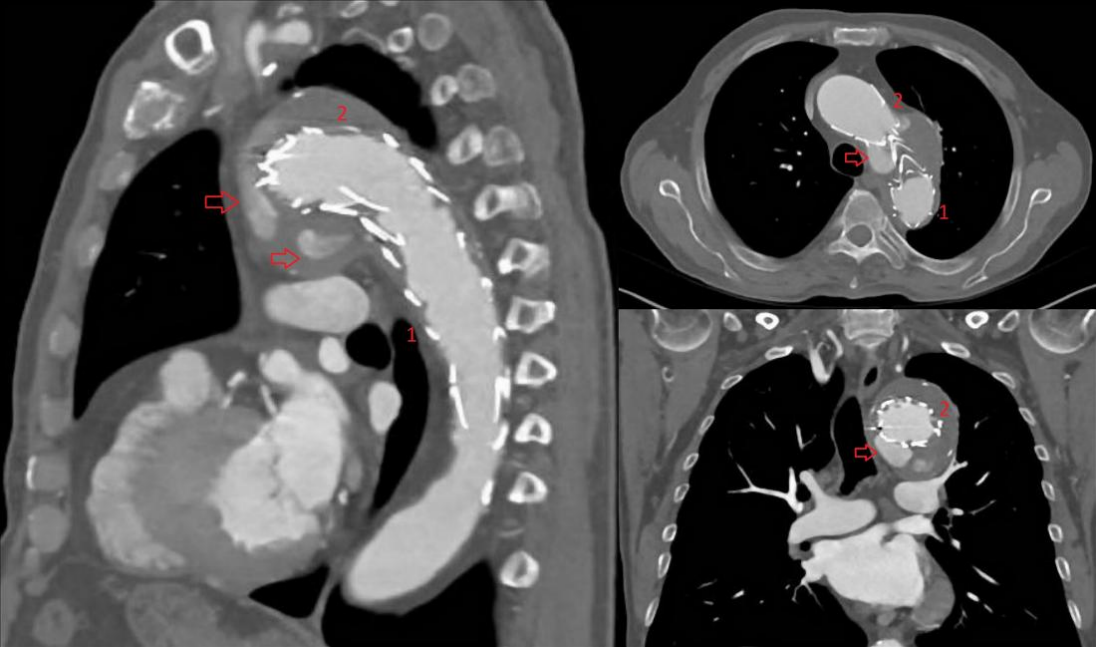
CT-scan showing a dilated aortic arch 65 × 67 mm due to persistent type 1A endoleak in zone 2, arrows. First TEVAR Medtronic Valiant Captivia 36 × 32 × 150 mm with proximal landing in zone 3, number 1; Second TEVAR Medtronic Valiant Captivia 38 × 38 × 100 mm with proximal landing in zone 2, number 2.

We describe a case of a 90-year-old man admitted with a diagnosis of severe aortic stenosis and persistent type-1A endoleak post-thoracic endovascular aortic repair (TEVAR). Previously, the patient underwent TEVAR due to a complicated type-B aortic dissection (Medtronic Valiant Captivia 36 × 32 × 150 mm). Afterwards, he experienced a type-1A endoleak and progressive dilatation of the proximal descending thoracic aorta. Therefore, he was scheduled for concomitant left-subclavian to the left-carotid artery transposition and TEVAR. A Medtronic Valiant Captivia 38 × 38 × 100 mm endoprosthesis was implanted with the proximal landing zone in zone 2. Unfortunately, a persistent type-1A endoleak was associated with a further dilatation of the aortic arch. A computed tomography (CT) scan showed a dilated aortic arch of 65 × 67 mm diameter (*Figure 1*). In addition, a known aortic valve stenosis progressed until a severe degree. A pre-operative transthoracic echocardiogram showed a normal functioning left ventricle with mild hypertrophy, calcified tricuspid aortic valve with aortic valve area at 0.94 cm^2^ and mean gradient at 39 mmHg, ascending aorta at 37 mm diameter. Considering the age of the patient and his comorbidities (Euroscore II 13.70%), after discussion in a multidisciplinary meeting, it was agreed for a hybrid approach combining a transcatheter aortic valve replacement (TAVR) with a brachiocephalic trunk and left common carotid artery debranching and TEVAR of the ascending aorta and arch.

**Figure 2:**
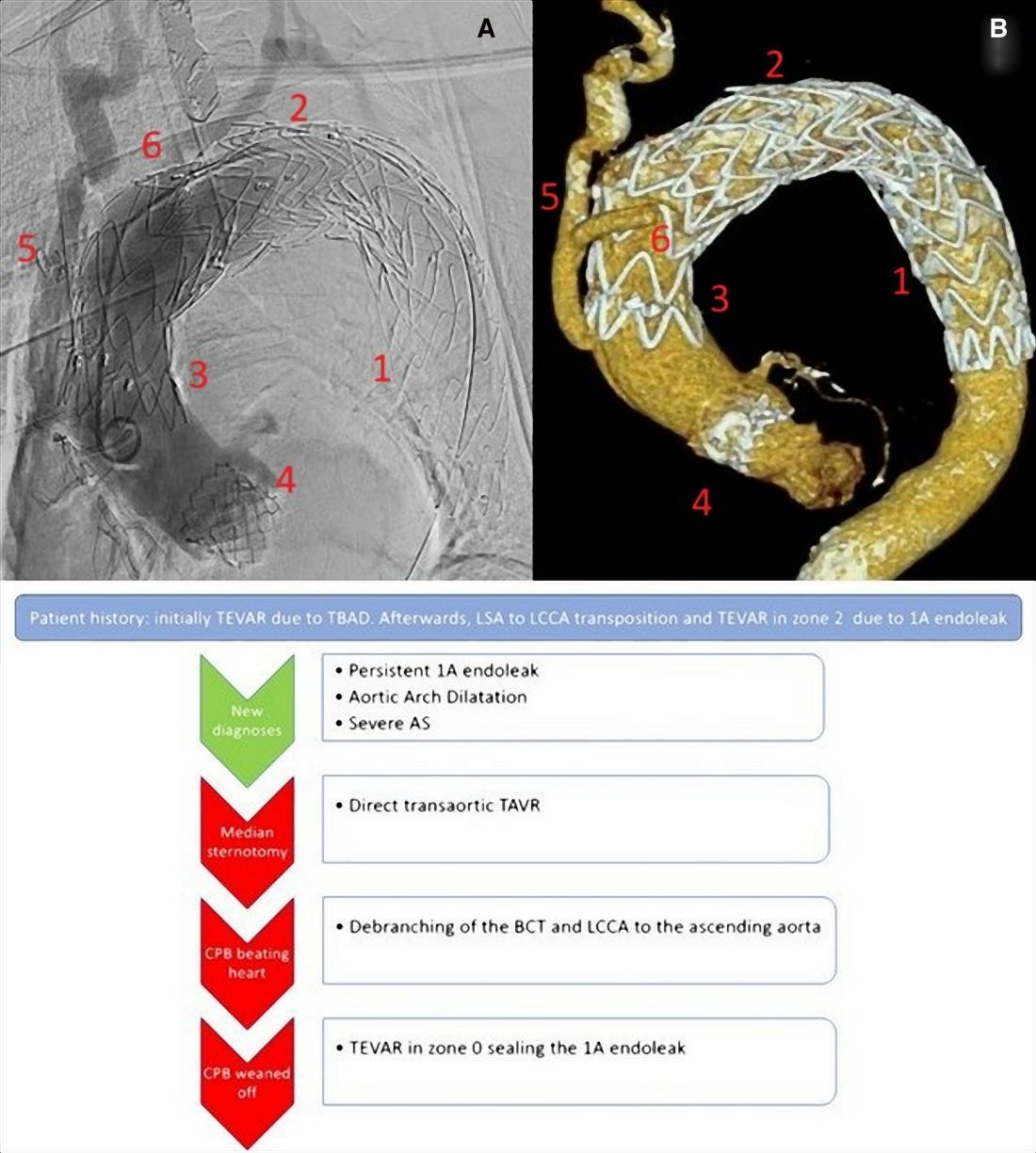
(*A*) angiography and (*B*) CT-scan 3D reconstruction post procedure and below a diagram with a step by step procedure description; 1: first TEVAR landing in zone 3; 2: second TEVAR landing in zone 2; 3: third TEVAR landing in zone 0; 4: TAVR; 5: 10 mm vascular prosthesis from the ascending aorta to the brachiocephalic trunk; 6: 8 mm vascular prosthesis from the previous 10 mm prosthesis to the left common carotid artery.

A median sternotomy was performed and the cardiopulmonary bypass (CPB) was instituted through the right-subclavian artery and the right atrium. A direct transaortic TAVR was performed with a 26 mm Sapiens 3 Ultra. Then, a debranching of the brachiocephalic trunk and the left common carotid artery was performed under CPB assistance on a beating heart. A side clamp was applied at the ascending aorta and the anastomosis of a 10 mm vascular prosthesis was performed with Prolene 4-0. An end-to-end anastomosis of a 8 mm vascular prosthesis to the left common carotid artery was performed with Prolene 4-0. Then, an end-to-side anastomosis of the 8 mm to the 10 mm vascular prosthesis was performed with Prolene 4-0. Finally, an end-to-end anastomosis of the 10 mm vascular graft was performed to the brachiocephalic trunk. The CPB was weaned and the arterial and venous cannula removed. The procedure was completed performing a TEVAR through the right common femoral artery. An endoprosthesis, Medtronic Captivia 40 × 40 × 200 mm, was delivered in the arch landing in zone 0 and overlapped with the previous endoprosthesis in zone 2 in order to seal the type-1A endoleak achieving a good angiographic result (*Figure 2A*).

The postoperative recovery was uneventful and the patient was discharged to an inpatient rehabilitation facility at postoperative day 15. A pre-discharge CT scan confirmed the successful procedure without residual endoleak (*Figure 2B*).

## Discussion

Complex aortic surgery carries higher operative risk when performed in nonagenarians and evidence in literature is scarce and based on case reports or small series. A recent multicentre retrospective observational study identified 18 nonagenarians who underwent valve surgery, CABG or a combination of them via a median sternotomy over an 8-year period. The median EuroSCORE-II predicted in-hospital mortality was 6.1% and at 6 months after surgery all patients were alive showing that a multidisciplinary patient selection and dedicated peri-operative care can lead to acceptable outcomes.^1^ Nasso *et al*. compared their results operating on nonagenarians with ‘open’ cardiac surgery (‘on’ or ‘off’ pump for coronary surgery; conventional or minimally invasive for valve surgery) from 2009 to 2021 (101 patients) and from 1998 to 2008 (127 patients). Their 30-day mortality improved from 13.4% to 5.9% in the more recent cohort of patients demonstrating acceptable risks in elderly patients.^2^ Elsisy *et al*. operated on 134 patients at 90 years of age or older that underwent isolated CABG, combined CABG and valve replacement or repair and isolated valve replacement or repair between 1993 and 2019. Their operative mortality rate was 6%.^3^ The experience of Ikeno *et al*. operating on 139 patients aged 80 and more of total arch replacement (72.7% elective surgery, 27.3% urgent or emergency surgery) showed an operative mortality at 8.6% and 1-year mortality at 27.2%. The authors concluded that total arch replacement was performed with acceptable survival. However, concomitant procedures significantly increased late-term mortality.^4^ Yamamoto *et al*. performed a successful combined arch debranching and TEVAR due to an aortic arch aneurysm in a 94-year-old woman.^5^

We described for the first time a scenario of a nonagenarian with complex history of aortic disease who presented a concomitant dilated aortic arch at 67 mm and a severe aortic stenosis. We thought that a conventional open aortic arch surgery with hypothermic cardiac arrest and concomitant aortic valve replacement would have increased the operative risk excessively. The meaningful preoperative multidisciplinary discussion with cardiac surgeons, interventional cardiologists and interventional radiologists was in favour of a hybrid strategy to reduce the overall procedural risk.

## Data Availability

The data underlying this article will be shared on reasonable request to the corresponding author.

## Lead author biography

Mr Mirko Muretti is a Cardiac Surgeon who is working at Istituto Cardiocentro Ticino, Switzerland. He started is training at Istituto Clinico Humanitas in Milan, Italy. He had a fellowship in one of the largest heart centres in the Netherlands, the St. Antonius Hospital. Then he moved to the United Kingdom where he worked at centres with international reputation such as the Royal Brompton Hospital in London; Oxford University Hospital in Oxford; The James Cook University Hospital in Middlesbrough and Royal Sussex County Hospital in Brighton. His main area of interest is minimally invasive surgery.

## Notes

**Consent:** Consent for publication has been obtained, in line with the COPE best practice guidelines, and that the individual(s) who is being reported on is aware of the possible consequences of that reporting.
